# Relevance of cyclin D1b expression and *CCND1 *polymorphism in the pathogenesis of multiple myeloma and mantle cell lymphoma

**DOI:** 10.1186/1471-2407-6-238

**Published:** 2006-10-06

**Authors:** Sophie Krieger, Juliette Gauduchon, Mikel Roussel, Xavier Troussard, Brigitte Sola

**Affiliations:** 1 Laboratoire de Biologie Moléculaire et Cellulaire de la Signalisation, EA 3919, Université de Caen, France; 2 Laboratoire d'Hématologie, CHU Côte de Nacre, Caen, France

## Abstract

**Background:**

The *CCND1 *gene generates two mRNAs (cyclin D1a and D1b) through an alternative splicing at the site of a common A/G polymorphism. Cyclin D1a and b proteins differ in their C-terminus, a region involved in protein degradation and sub-cellular localization. Recent data have suggested that cyclin D1b could be a nuclear oncogene. The presence of cyclin D1b mRNA and protein has been studied in two hemopathies in which cyclin D1 could be present: multiple myeloma (MM) and mantle cell lymphoma (MCL). The A/G polymorphism of *CCND1 *has also been verified in a series of patients.

**Methods:**

The expression of cyclin D1 mRNA isoforms has been studied by real-time quantitative PCR; protein isoforms expression, localization and degradation by western blotting. The *CCND1 *polymorphism was analyzed after sequencing genomic DNA.

**Results:**

Cyclin D1 mRNA isoforms a and b were expressed in mantle cell lymphoma (MCL) and multiple myeloma (MM). Cyclin D1b proteins were present in MCL, rarely in MM. Importantly, both protein isoforms localized the nuclear and cytoplasmic compartments. They displayed the same short half-life. Thus, the two properties of cyclin D1b recognized as necessary for its transforming activity are missing in MCL. Moreover, *CCND1 *polymorphism at the exon/intron boundary had no influence on splicing regulation in MCL cells.

**Conclusion:**

Our results support the notion that cyclin D1b is not crucial for the pathogenesis of MCL and MM.

## Background

Cyclin D1 is a critical regulator of the cell cycle and transcriptional processes. Overexpression or overactivity of cyclin D1 is common in human cancers. Cyclin D1 is also expressed in lymphoid tumors such as mantle cell lymphoma (MCL) and multiple myeloma (MM). The expression of cyclin D1 in B-cells in which the protein is physiologically absent, is supposed to be causal in the transformation process. In MCL, *CCND1 *(encoding cyclin D1) is activated by the t(11;14)(q13;q32) in almost 100% of cases [1]. In multiple myeloma (MM), cyclin D1 is expressed in 45–50% of samples but the t(11;14)(q13;q32) is only present in 15% of them [2]. The human *CCND1 *gene encodes two mRNAs species resulting from an alternative splicing: form a [3] and form b generated by the absence of splicing at the exon 4/intron 4 boundary [4]. Cyclin D1 protein isoforms differ in the last 55 amino acids of the carboxy-terminus. Cyclin D1a and D1b proteins possess the cyclin box necessary for cyclin-dependent kinase (CDK) binding and enzymatic activity. But the PEST destruction box as well as Thr286, the phosphorylation site of glycogen synthase kinase-3β which promotes the nuclear export of cyclin D1 and its turn-over, are missing in form b [5,6]. Recently, it has been shown that cyclin D1 isoforms could display functional differences and importantly that only cyclin D1b facilitates transformation in fibroblasts [7-9]. Considering this putative role in tumorigenesis, we have studied cyclin D1b expression in MCL and MM samples. A common A/G polymorphism described as a modulator of cancer risk is thought to regulate the production of cyclin D1b [4]. We have also analyzed this relationship in MCL samples.

## Methods

### MCL and MM patients and cell lines

Patients with MCL or MM were diagnosed according to criteria from the World Health Organization classification in our institution (CHU Côte de Nacre, Caen, France); their clinical features are presented in the Additional file 1. Signed informed consent was obtained from all patients before all procedures as required by the institutional review board. Peripheral blood or bone marrow samples were obtained at diagnosis. Mononuclear cells were isolated by centrifugation over a Ficoll-Hypaque layer. CD19+ cells from 10 MCL patients were then purified (>90%) using a MACS microbeads system (Miltenyi Biotechnology). CD138+ cells from 6 MM patients were purified with the same system (purity >85%). JeKo-1, NCEB-1, HBL-2 and GRANTA-519 are MCL cell lines. Karpas 620, U266, NCI-H929, RPMI 8226, OPM-2 are MM cell lines. As previously reported by us, they all expressed cyclin D1 except OPM-2. Cell lines were maintained in RPMI 1640 medium supplemented with 2 mM L-glutamine, 10% heat-inactivated fetal calf serum and antibiotics.

### Real-time quantitative RT-PCR

Total RNA was isolated from purified MCL and MM cells and cell lines with the RNeasy Micro Kit (QIAGEN S.A.) and reverse-transcribed with the 1^st ^Strand cDNA Synthesis kit (Roche). Real-time quantitative RT-PCR was performed on an ABI PRISM 7700 Sequence Detector with the SYBR^® ^Green PCR Master mix (Applied Biosystems). Primers were designed with the ABI PRISM Primer Express^® ^software; their sequences are presented in Table 1. For MCL and MM samples, RT-PCR amplifications were performed in a final volume of 15 μl containing PCR master mix, 5 ng of cDNA equivalents and primers (300 nM each for 18S rRNA, 400 nM for cyclin D1a and 800 nM for cyclin D1b). As negative control, real-time PCR was performed with purified non reverse-transcribed RNA of each sample. Expression of cyclin D1 isoforms compared to 18S rRNA was evaluated with the comparative threshold method (Ct). Each experiment was done in triplicate, triplicate values were averaged and averaged Ct values for 18S rRNA were subtracted from each value to give the ΔCt. U266 and JeKo-1 cells expressing high levels of both cyclin D1a and D1b mRNA were chosen as calibrators for MM and MCL cells, respectively. ΔCt obtained from U266 or JeKo-1 was subtracted from the ΔCt of each cell line to give ΔΔCt. The relative amount of each cyclin D1 isoform compared to the calibrator was calculated by the formula N = 2^-ΔΔCt^.

### Genomic DNA purification, cDNA synthesis and sequencing

Genomic DNA of CD19+ MCL cells was isolated with the Wizard^® ^Genomic DNA (Promega). Total RNA was extracted from purified MCL cells and reverse-transcribed as before. Genomic DNA and cDNA were used as templates for PCR amplification using the Exon4 D1F and Intron4 D1R primers (see sequences in Table 1). PCR-amplified fragments encompassing the exon 4/intron 4 boundary were sequenced using the Big Dye Terminator Sequencing kit (Applied Biosystems). Sequences were analyzed with the ABI PRISM 310 Genetic Analyzer.

### Immunoblotting

Nuclear, cytoplasmic or whole cell extracts, SDS-PAGE, electro-transfer and immunoblotting, all are procedures described previously [10]. The DCS-6 monoclonal anti-cyclin D1 antibody (Ab, Pharmingen BD Biosciences n°556070) is directed against the entire cyclin D1 molecule and recognizes both isoforms; the sc-718 anti-cyclin D1 Ab (Santa Cruz Biotechnologies) is directed against the C-terminal part of the protein and is specific for cyclin D1a, the R3 Ab is directed against the C-terminal part of cyclin D1b and is specific for this isoform [7]. The R3 Ab was a generous gift of Dr Alan J. Diehl. Reprobing blots with anti-β-tubulin Ab (sc-9104, Santa Cruz Biotech.) or anti-E2F1 (sc-251, Santa Cruz Biotech.) allowed us to check protein loading and transfer. Densitometric analyses were realized with a FluorSImager using the QuantityOne software (Bio-Rad).

## Results

### Expression of cyclin D1 mRNA isoforms in MCL and MM patients and cell lines

**Figure 1:**
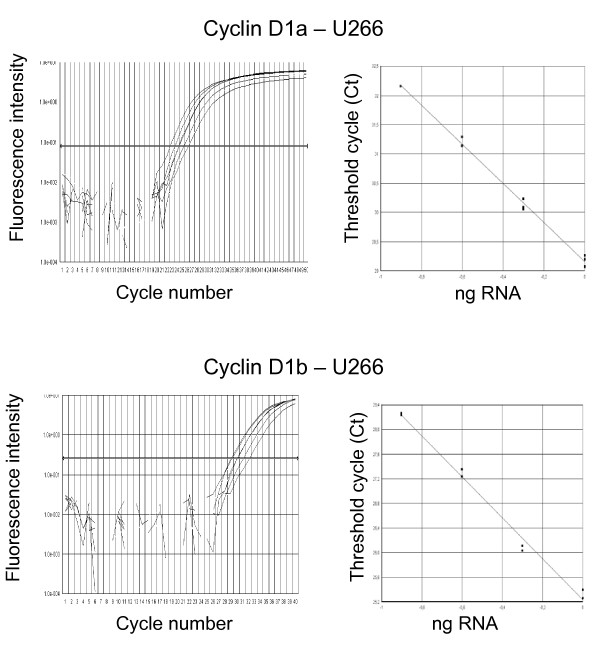
**Real-time RT-PCR assay for cyclin D1a and b expression**. Total RNA isolated from U266 MM cell line was serially diluted to obtain dilutions ranging from 10 to 0.625 ng. Cyclin D1a was amplified by RT-PCR using QRT D1a S and QRT D1a AS primers (400 nM) and cyclin D1b using QRT D1b S and QRT D1b AS primers (800 nM, Table 1). Amplifications are shown in logarithmic scale (left part). Threshold cycle (Ct) was plotted against quantity (ng) of RNA to obtain standard curve (right part). The slope of the curve is -3.32 indicating an amplification efficiency of 100%

Both spliced mRNAs have been detected by standard RT-PCR in MCL primary cells and MM cell lines [11]. To quantify the relative amounts of mRNA isoforms, primers specific for each isoform were designed and real-time quantitative RT-PCR was performed. The reliability of the method is illustrated in Figure 1. Total RNA purified from U266 cells was reverse-transcribed and serially diluted and samples were then amplified using the specific primers. A linear correlation was confirmed by the plot of input amount of RNA (X-axis) *vs*. Ct values (Y-axis) with minimal deviation among duplicates or triplicates. The efficacy of amplification was 100% for cyclin D1a and cyclin D1b allowing the comparison of their relative levels. The expression of cyclin D1 transcripts was normalized to 18S rRNA as internal standard. As expected, cyclin D1a mRNA was detected in all MCL patients with ΔCta varying from 8.8 to 12.9 (Table 2). In 9 out of 10 MCL patients, we found the co-expression of isoform b with ΔCtb varying from 14.3 to 19.3. MCL patient 3 expressed only the isoform a. Cyclin D1a was predominant over cyclin D1b with a ratio R varying from 33 to 80 (Table 3). In MCL cell lines, isoform levels were almost in the same range than in primary cells (ΔCta 6.5–12.5, ΔCtb 12.7–19.7). In only 3 out of 6 MM patients, total cyclin D1 was detected in CD138+ purified cells (data not shown); these three patients were studied further for cyclin D1 isoform expression. As expected, in MM primary cells and cell lines, cyclin D1 a and b expression was highly heterogeneous (ΔCta 10.0–18.2, ΔCtb 18.5–27.7) but perfectly correlated. They globally expressed less cyclin D1b mRNA than MCL (Table 2). The calculation of N illustrates the heterogeneity of cyclin D1a and b expression inside each series of samples (MM *vs*. MCL) and the heterogeneity of cyclin D1a *vs*. cyclin D1b expression for each case (see Additional file 2).

### Expression, localization and stability of cyclin D1b protein in MCL samples

**Figure 2:**
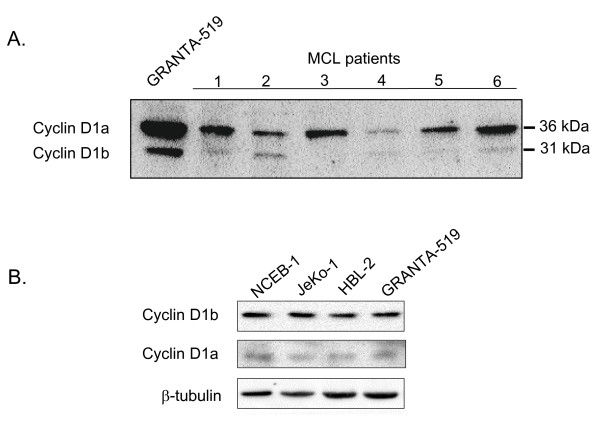
**Cyclin D1a and cyclin D1b protein expression**. A. Whole cell extracts were obtained from CD19+ cells of MCL patients, separated on SDS-PAGE and sequentially immunoblotted with anti-cyclin D1a (sc-718) or anti-cyclin D1b (R3) Abs. For loading controls, blots were also revealed with anti-β-tubulin Ab (sc-9104, not shown). B. Proteins were obtained from MCL cell lines and analyzed as described in A except that we used DCS-6 anti-cyclin D1 Ab which recognizes both isoforms.

**Figure 3:**
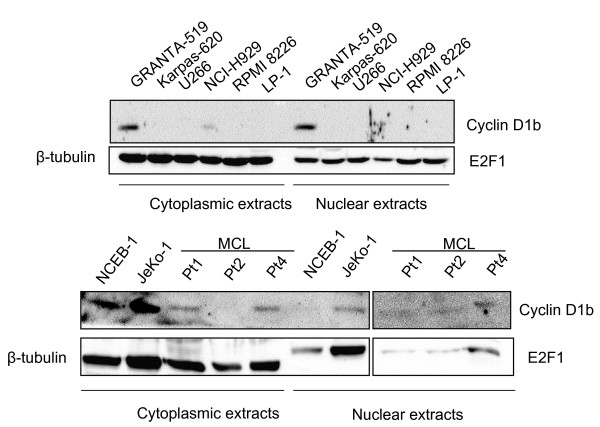
**Cyclin D1b sub-cellular localization and stability**. Nuclear and cytoplasmic extracts were prepared from cell lines and purified CD19+ primary cells and analyzed as described before. Blots were re-probed with anti-β-tubulin or anti-E2F1 (nuclear extracts, sc-251) Abs for loading controls.

**Figure 4:**
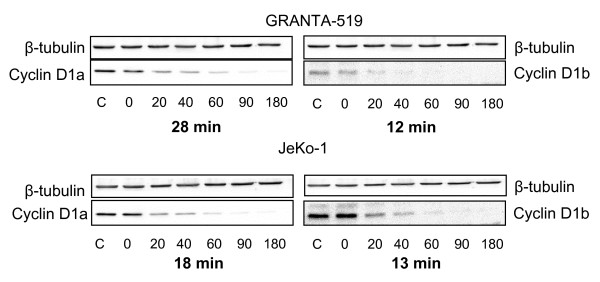
**Stability of cyclin D1a and cyclin D1b isoforms**. GRANTA-519 and JeKo-1 cells were untreated (C) or treated with cycloheximide (50 μg/ml final concentration) for 3 h. Cells were harvested 20–180 min later and total proteins were extracted and analyzed as described before. A densitometric analysis was performed to quantify proteins level and to estimate proteins half-life. Half-lives are indicated under the blots.

We detected the presence of both protein isoforms in MCL samples except for patient 3 (Figure 2A) and in cell lines (Figure 2B). The protein level of cyclin D1a (estimated by densitometric analysis) was higher than of form b (from 2 to 15-fold). In whole cell extracts, cyclin D1b was not detected in MM cell lines (data not shown), in good agreement with previous report [11]. Since cyclin D1 isoforms could display different sub-cellular localizations, nuclear and cytoplasmic protein extracts were prepared from MCL cell lines and primary cells and from MM cell lines, then immunoblotted. In MCL cell lines and primary cells, cyclin D1a (data not shown) and cyclin D1b localized both the nuclear and the cytoplasmic compartments with the exception of Pt 2 for which cyclin D1b was only nuclear (Figure 3). A very faint band was also visible in both extracts in NCI-H929 MM cells. Although not having the nuclear export signal, cyclin D1b displays the same sub-cellular distribution than cyclin D1a. GRANTA-519 and JeKo-1 cell lines were treated with cycloheximide to inhibit protein synthesis. We then monitored protein stability at the times indicated in the Figure 4. Cyclin D1b exhibited a half-life similar to cyclin D1a in both cell lines (indicated under each immunoblot). Both are short-lived proteins despite the absence of PEST sequences in isoform b.

### Analysis of *CCND1 *polymorphism at position 9630 of genomic DNA and at position 870 of mRNA – Relation with the level of cyclin D1 forms a and b

*CCND1 *alternative splice could be modulated by a common G870A polymorphism within the splice donor site [4]. Moreover, AA genotype could be associated with a preferential expression of isoform b and a higher risk of cancers [12]. As presented in Table 3, MCL patient 5 was homozygous for the A allele, patient 6 homozygous for the G allele, the other 4 were heterozygous. Each tumor expressed either the A allele (3/6) or the G allele (3/6) but none expressed both alleles. Moreover, cyclin D1b was transcribed from the A or G allele and whatever the nucleotide at position 870, cyclin D1a was more expressed than cyclin D1b. The A/G genotype does not influence the relative level of the two transcripts.

## Discussion

Using standard RT-PCR, we found previously that cyclin D1a and b were coexpressed in MCL patients and in MM cell lines irrespectively of the presence of the t(11;14) translocation [11]. We now extended this work and analyzed cyclin D1 isoform expression with a real-time quantitative RT-PCR in MM and MCL primary cells and cell lines.

Although cyclin D1 mRNA isoforms are always co-expressed in MCL and in cyclin D1-expressing MM cells, cyclin D1b protein was rarely and faintly expressed in MM samples. By contrast, in MCL cells, cyclin D1b was often present. Thus, post-transcriptional mechanisms regulate cyclin D1b status depending on the B-cell hemopathy.

Cyclin D1a and D1b proteins exhibit the same stability. In previous studies [7,8], in transfected models, the calculated half-lives of cyclin D isoforms were also similar and similar to ours. Thus, endogenous and exogenous cyclin D1 isoforms are subjected to the same post-translational regulation. Either the PEST sequence is not strictly necessary for proteolysis through the proteasome machinery which is peculiarly active in MCL [13] or cyclin D1 can be degraded through another motif. In the case of cell response to stress, cyclin D1 can be degraded through its binding to the anaphase-promoting complex and a RXXL sequence located in the NH2-terminal part of the protein [14]. This sequence is present in both cyclin D1 isoforms. Such a mechanism could be activated constitutively in MCL cells.

B-cell lymphomas can be induced in transgenic mice expressing a cyclin D1 mutant (T286A) under the control of Eμ enhancer [15]. This mutant displays a 5-fold longer half-life than cyclin D1 [16] and is exclusively nuclear. This sub-cellular localization seems to be a prerequisite to transformation. In MCL cells, cyclin D1b localizes both the nuclear and cytoplasmic compartments. In good agreement, endogenous cyclin D1b appears cytoplasmic and nuclear in oesophageal carcinomas [7] In contrast, in transfected fibroblastic cells, cyclin D1b remains mostly nuclear [7-9]. The simplest explanation is that the nuclear export is subverted by an overexpression of exogenous cyclin D1b.

Cyclin D1b is rarely and faintly expressed in MM and not always detected in MCL ([17] and our results). Thus, the expression of cyclin D1b is not necessary at least for the maintenance of a tumoral phenotype. Interestingly, the presence of a short cyclin D1 transcript (1.7 kb) lacking the 3'-UTR region responsible for mRNA stability, has been associated with MCL aggressiveness and a poor prognosis [1]. The relationship between the alternative spliced forms and the short mRNA has to be studied.

In MCL, *CCND1 *alternative splicing does not modulate the level of transcripts b. This is consistent with results showing that *CCND1 *alternative splicing depends on sample origin [17]. Moreover, cyclin D1b is transcribed independently of A/G genotype. This indicates that both alleles can splice to form both transcripts. Besides A/G polymorphism, *trans*-elements regulate alternative splicing. In good agreement with us, Howe and Lynas also showed that *CCND1 *polymorphism does not affect the prognosis of MCL patients [18].

In breast, sarcoma and colon cancers, with cyclin D1 overexpression and no chromosome 11 alterations, elevated mRNA levels result from a *trans*-acting influence of both alleles [19]. A biallelic expression is seen in MM tumors without the t(11;14) [2]. In agreement with that observed in MM tumors with t(11;14), only one allele is transcribed in MCL cells, the transcribed allele is likely the structurally altered one.

## Conclusion

Although cyclin D1b mRNA is produced in the vast majority of MM and MCL cells, cyclin D1b protein is not always found and not found exclusively in the nucleus. These data allowed us to conclude that a transforming activity of cyclin D1b over cyclin D1a is unlikely. Moreover, our data establish no relationship between A/G polymorphism and cyclin D1b expression. The relevance of cyclin D1b in MCL and MM pathologies remains elusive.

## Competing interests

The author(s) declare that they have no competing interests.

## Authors' contributions

SK performed most experiments and JG the others. SK, JG, MR contributed to analysis and interpretation of data and to approval of the manuscript. XT provided patient samples, critically revised the manuscript and approved the manuscript. BS designed experiments, participated to analysis, interpretation of data, wrote the two versions of the paper. All authors read and approved the version 2 of the manuscript.

## Pre-publication history

The pre-publication history for this paper can be accessed here:



## Supplementary Material

Additional file 1 Clinical features of MCL and MM patients. The table provides some clinical parameters from patients studied (age, sex, blood numeration and formula, karyotype etc.). Click here for file

Additional file 2 Real-time RT-PCR analysis of cyclin D1a and b mRNA levels in MCL and MM patients. The figure provides the data obtained after real-time RT-PCR analysis of cyclin D1a and b mRNA levels in MCL (left panel) and MM right panel patients. According to the comparative threshold method (Ct) using 18S rRNA as internal standard and by referring to an internal calibrator (JeKo-1/MCL and U266/MM), we calculated the relative amount N of cyclin D1a and b by the formula N = 2^-ΔΔCt^. Click here for file

## Figures and Tables

**Table 1:** Sequences and positions of primers

Primer	Position	Sequence
QRT D1a S	1139 (M64349)*	5'-GGA AAG CTT CAT TCT CCT TGT TG-3'
QRT D1a AS	1217 (M64349)	5'-TTC TTT TGC TTA AGT CAG AGA TGG AA-3'
QRT D1b S	294 (X88930)	5'-GCC AAT GGT CTG TGT GGT GAT-3'
QRT D1b AS	371 (X88930)	5'-ATT GGC CAC GCA CAT TGT G-3'
QRT 18S S	450 (X03205.1)	5'-CGG CTA CCA CAT CCA AGG AA-3'
QRT 18S AS	636 (X03205.1)	5'-GCT GGA ATT ACC GCG GCT-3'

Exon4 D1F	719 (M64349)	5'-ATG TGA AGT TCA TTT CCA ATC C-3'
Intron4 D1R	195 (X88930)	5'-CCA GTC AGT AAG TTC TAG GAG CAG-3'

* Accession numbers in GenBank are in brackets.

**Table 2:** Cyclin D1a and cyclin D1b mRNA expression in MCL and MM samples

Sample	ΔCta	ΔΔCta	Na	ΔCtb	ΔΔCtb	Nb
**MCL**						
JeKo-1*	6.5	0	1	12.7	0	1
GRANTA-519	12.5	6.0	0.0156	19.0	6.3	0.0127
HBL-2	7.8	1.3	0.4061	14.5	1.8	0.2871
NCEB-1	10.9	4.4	0.0474	19.7	7.0	0.0078
Pt 1	10.4	3.9	0.0670	19.3	6.6	0.0103
Pt 2	11.3	4.8	0.0359	16.4	6.7	0.0096
Pt 3	10.1	5.6	0.0206	nc	nc	nc
Pt 4	12.9	6.4	0.0118	18.2	8.5	0.0028
Pt 5	9.2	2.7	0.1539	15.5	5.8	0.0179
Pt 6	8.8	2.3	0.2030	15.0	5.3	0.0254
Pt 7	11.4	4.9	0.0335	16.9	7.2	0.0068
Pt 8	9.1	2.6	0.1650	14.3	4.6	0.0412
Pt 9	12.2	5.7	0.0192	17.0	7.3	0.0063
**MM**						
U266*	10.0	0	1	18.8	0	1
Karpas 620	13.9	3.9	0.0670	20.8	2.0	0.2500
RPMI 8226	17.1	7.1	0.0073	23.9	5.1	0.0291
NCI-H929	15.9	5.9	0.0167	23.7	4.9	0.0335
OPM-2	nc	nc	nc	nc	nc	nc
Pt 1	12.3	2.3	0.2030	18.5	-0.3	1.2311
Pt 2	18.2	8.2	0.0034	27.7	8.9	0.0021
Pt 3	15.0	5.0	0.0312	27.1	8.3	0.0032

The quantitation of cyclin D1a and cyclin D1b mRNA compared to 18S rRNA was done using the comparative threshold (Ct) method with the ABI PRISM 7000 SDS software. Triplicate experiments were done for each sample; for each sample the average Ct value for the internal standard was subtracted from the average Ct value for cyclin D1a or cyclin D1b to yield ΔCta and ΔCtb. ΔCt obtained from the calibrator cell lines (* JeKo-1 for MCL samples and U266 for MM samples) was subtracted from each ΔCta or ΔCtb to give the ΔΔCt. The relative amount of cyclin D1a and cyclin D1b compared to the calibrator was calculated by the formula N = 2^-ΔΔCt^. No cyclin D1b was detected in MCL patient 3 (Ctb>50); no cyclin D1a or b was detected in OPM-2 MM cell line (Cta>50 and Ctb>50). nc, not calculated; Pt, patient.

**Table 3:** *CCND1 *polymorphism, monoallelic transcription and cyclin D1 mRNA level

MCL patient	Position 9630	Position 870	R*
1	A/G	G	60
2	A/G	G	33
4	nd	nd	39
5	A	A	80
6	G	G	78
7	A/G	A	45
8	A/G	A	36

Genomic DNA and total RNA were extracted from CD19+ MCL cells and purified. RNA was reverse-transcribed and DNA samples were PCR-amplified before sequencing with Exon4 D1F and Intron4 D1R primers. Only the cyclin D1b mRNA form can be amplified with this couple of primers. Nucleotides at position 9630 (AP001888, GenBank) of genomic DNA and at position 870 of cDNA (M64349, GenBank) are indicated. * R, the relative ratio of cyclin D1a mRNA *vs*. cyclin D1b mRNA was calculated with the formula R = 2^-(ΔCta-ΔCtb)^. nd, not determined.

## References

[B1] Rosenwald A, Wright G, Wiestner A, Chan WC, Connors JM, Campo E, gascoyne RD, Grogan TM, Muller-Hermelink HK, Smeland EB, Chiorazzi M, Giltnane JM, Hurt EM, Zhao H, Averett L, Henrickson S, Yang L, Powell J, Wilson WH, Jaffe ES, Simon R, Klausner RD, Montserrat E, Bosch F, Greiner TC, Weisenburger DD, Sanger WG, Dave BJ, Lynch JC, Vose J, Armitage JO, Fisher RI, Miller TP, LeBlanc M, Ott G, Kvaloy S, Holte H, Delabie J, Staudt LM (2003). The proliferation gene signature is a quantitative integrator of oncogenic events that predicts survival in mantle cell lymphoma. Cancer Cell.

[B2] Bergsagel PL, Kuehl WM, Zhan F, Sawyer J, Barlogie B, Shaughnessy J (2005). Cyclin D dysregulation: an early and unifying pathogenic event in multiple myeloma. Blood.

[B3] Inaba T, Matsushime H, Valentine M, Roussel MF, Sherr CJ, Look AT (1992). Genomic organization, chromosomal localization, and independent expression of human cyclin D genes. Genomics.

[B4] Betticher C, Thatcher N, Altermatt HJ, Hoban P, Ryder WD, Heighway J (1995). Alternate splicing produces a novel cyclin D1 transcript. Oncogene.

[B5] Diehl JA, Cheng M, Roussel MF, Sherr CJ (1998). Glycogen synthase kinase-3beta regulates cyclin D1 proteolysis and subcellular localization. Genes Dev.

[B6] Alt JR, Cleveland JL, Hannink M, Diehl JA (2000). Phosphorylation-dependent regulation of cyclin D1 nuclear export and cyclin D1-dependent cellular transformation. Genes Dev.

[B7] Lu F, Gladden AB, Diehl JA (2003). An alternative spliced cyclin D1 isoform, cyclin D1b, is a nuclear oncogenes. Cancer Res.

[B8] Solomon DA, Wang Y, Fox SR, Lambeck TC, Giesting S, Lan Z, Senderowicz AM, Conti CJ, Knudsen ES (2003). Cyclin D1 splice variants. J Biol Chem.

[B9] Holley SL, Heighway J, Hoban PR (2005). Induced expression of human CCND1 alternative transcripts in mouse Cyl-1 knockout fibroblasts highlights functional differences. Int J Cancer.

[B10] Duquesne F, Florent M, Roué G, Troussard X, Sola B (2001). Ectopic expression of cyclin D1 impairs the proliferation and enhances the apoptosis of a murine lymphoid cell line. Cell Death Different.

[B11] Roué G, Krieger S, Florent M, Roussel M, Duquesne F, Troussard X, Pichereau V, Sola B (2003). Expression of the two alternative [a] and [b] transcripts of CCND1 gene in cyclin D1-expressing-malignancies: relevance for the pathogenesis. Leukemia.

[B12] Knudsen KE, Diehl JA, Haiman CA, Knudsen ES (2006). Cyclin D1: polymorphism, aberrant splicing and cancer risk. Oncogene.

[B13] Bogner C, Peschel C, Decker T (2006). Targeting the proteasome in mantle cell lymphoma: a promising therapeutic approach. Leuk Lymphoma.

[B14] Agami R, Bernards R (2000). Distinct initiation and maintenance mechanisms cooperate to induce G1 cell cycle arrest in response to DNA damage. Cell.

[B15] Gladden AB, Woolery R, Aggarwal P, Wasik MA, Diehl JA (2006). Expression of constitutively nuclear cyclin D1 in murine lymphocytes induces B-cell lymphoma. Oncogene.

[B16] Diehl JA, Sherr CJ (1997). A dominant-negative cyclin D1 mutant prevents nuclear import of cyclin-dependent kinase (CDK4) and its phosphorylation by CDK-activating kinase. Mol Cell Biol.

[B17] Carrère N, Belaud-Rotureau M-A, Dubus P, de Mascarel A, Merlio JP (2005). The relative levels of cyclin D1a and D1b alternative transcripts in mantle cell lymphoma may depend more on sample origin than on CCND1 polymorphism. Haematologica.

[B18] Howe D, Lynas C (2001). The cyclin D1 alternative transcripts [a] and [b] are expressed in normal and malignant lymphocytes and their relative levels are influenced by the polymorphism at codon 241. Haematologica.

[B19] Hosokawa Y, Arnold A (1998). Mechanims of cyclin D1 (CCND1, PRAD1) overexpession in human cancer cells: analysis of allele-specific expression. Genes Chromosomes Cancer.

