# Supplementary material for: Relevance of cyclin D1b expression and CCND1 polymorphism in the pathogenesis of multiple myeloma and mantle cell lymphoma
Source: BMC Cancer. 2006 Oct 6;6:238. doi: 10.1186/1471-2407-6-238 (PMC1609182; doi:10.1186/1471-2407-6-238)
Supplement: Additional file 1 — Clinical features of MCL and MM patients. The table provides some clinical parameters from patients studied (age, sex, blood numeration and formula, karyotype etc.). [file 1471-2407-6-238-S1.doc]

Additional file 1 – Clinical features of MCL and MM patients

| MCL patient | 1 | 2 | 3 | 4 | 5 | 6 | 7 | 8 | 9 | 10 |
| --- | --- | --- | --- | --- | --- | --- | --- | --- | --- | --- |
| Age | 59 | 49 | 58 | 78 | 75 | 65 | 77 | 61 | 74 | 81 |
| Sex | M | M | M | M | M | M | M | M | M | F |
| Hb (g/dl) | 12.5 | 14.0 | 11.8 | 11.2 | 10.5 | 10.3 | 10.6 | 13.5 | 12.6 | 13.5 |
| Plt (G/l) | 52 | 292 | 30 | 108 | 185 | 122 | 67 | 258 | 187 | 196 |
| WBC (G/l) | 32.4 | 21 | 4.5 | 8 | 19.6 | 93.2 | 82.5 | 6 | 22.3 | 7.4 |
| % CD5/CD19+ | 65 | 84 | 77 | 55 | 71 | 91 | 95 | 61 | 65 | 70 |
| Light chain | λ | κ | κ | λ | κ | κ | λ | λ | λ | λ |
| FMC7 | + | - | + | + | + | + | + | + | + | + |
| Matutes score | 2 | 2 | 2 | 1 | 2 | 2 | 3 | 2 | 2 | 2 |
| Karyotype | nd | nd | t(11;14) | t(11;14) | nd | t(11;14) | t(11;14) | nd | nd | nd |

| MM patient | 1 | 2 | 3 | 4 | 5 | 6 |
| --- | --- | --- | --- | --- | --- | --- |
| Age | 68 | 54 | 77 | 72 | 56 | 56 |
| Sex | M | M | F | F | M | M |
| Hb (g/dl) | 7.2 | 15 | 6.8 | 13.6 | 11.6 | 14.0 |
| Plt (G/l) | 172 | 303 | 236 | 318 | 162 | 193 |
| WBC (G/l) | 4.9 | 9.1 | 4.4 | 7.2 | 4.1 | 4.0 |
| % plasmocytes | 32 | 10 | 32 | 11 | 57 | 57 |
| IgH/L | D/λ | G/κ | G/κ | A/κ | M/κ | A/λ |
| Cyclin D1 mRNA* | + | + | + | - | - | - |

*CD138+ purified cells from MM patients were analyzed by real-time quantitative RT-PCR using a couple of primers uncapable to discriminate between the two isoforms (their sequences are available upon request). Total cyclin D1 mRNA was detected only in patients 1, 2 and 3. Only these three samples were studied further for the presence of cyclin D isoforms. Abbreviations: Hb, hemoglobin; Plt, platelets; WBC; white blood cells; G, 10^9^; Ig, immunoglobulin; nd, not determined.
