# Supplementary figures and images for: Relevance of cyclin D1b expression and CCND1 polymorphism in the pathogenesis of multiple myeloma and mantle cell lymphoma
Source: BMC Cancer. 2006 Oct 6;6:238. doi: 10.1186/1471-2407-6-238 (PMC1609182; doi:10.1186/1471-2407-6-238)

## Slide 1
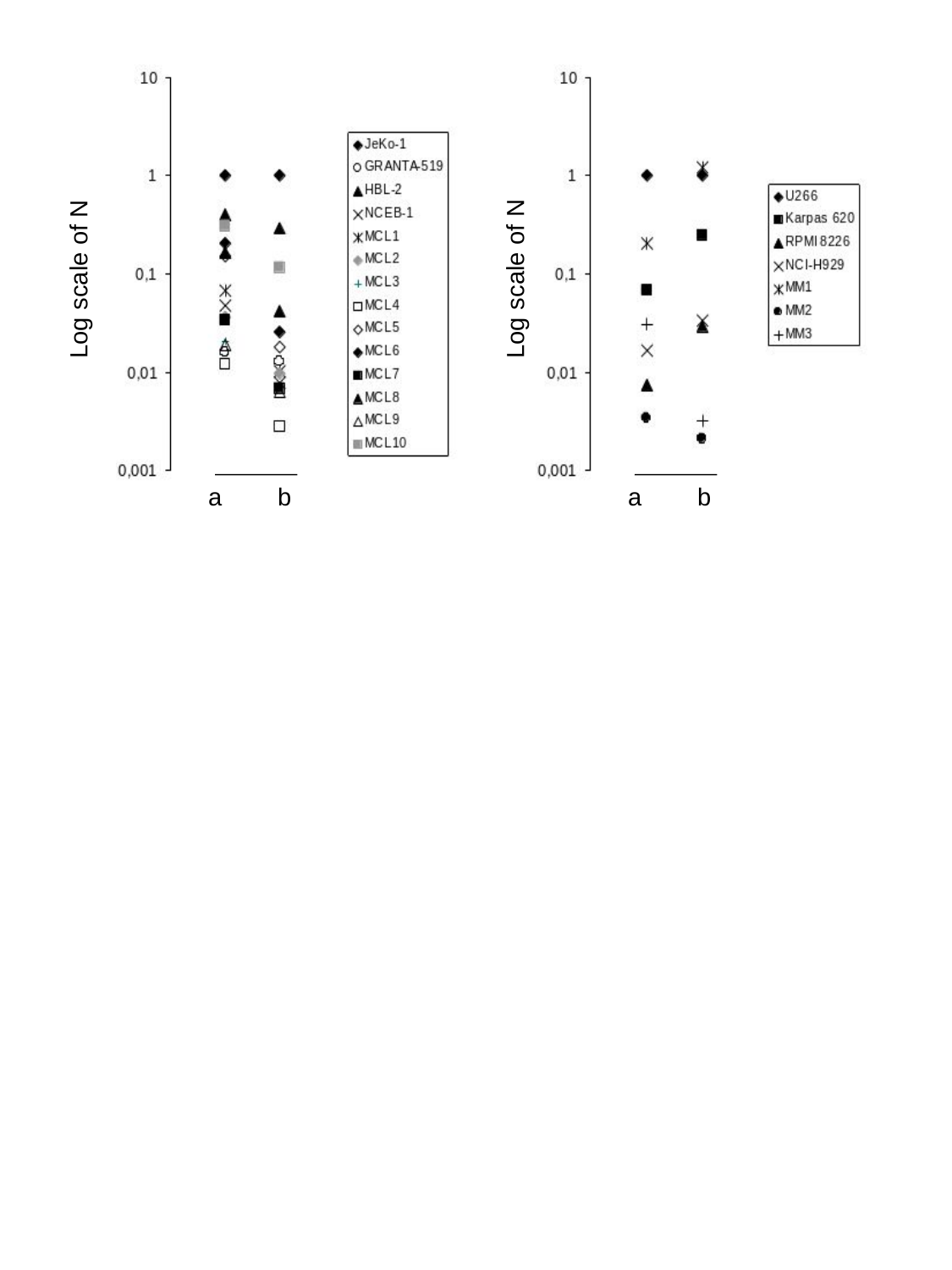

Log scale of N
Log scale of N
a b
a b

Supplement: Additional file 2 — Real-time RT-PCR analysis of cyclin D1a and b mRNA levels in MCL and MM patients. The figure provides the data obtained after real-time RT-PCR analysis of cyclin D1a and b mRNA levels in MCL (left panel) and MM right panel patients. According to the comparative threshold method (Ct) using 18S rRNA as internal standard and by referring to an internal calibrator (JeKo-1/MCL and U266/MM), we calculated the relative amount N of cyclin D1a and b by the formula N = 2-ΔΔCt. [file 1471-2407-6-238-S2.ppt]
